# The impact of type 2 diabetes on health related quality of life in Bangladesh: results from a matched study comparing treated cases with non-diabetic controls

**DOI:** 10.1186/s12955-016-0530-7

**Published:** 2016-09-13

**Authors:** Novie Safita, Sheikh Mohammed Shariful Islam, Clara K. Chow, Louis Niessen, Andreas Lechner, Rolf Holle, Michael Laxy

**Affiliations:** 1 Helmholtz Zentrum München, Institute of Health Economics and Health Care Management, Neuherberg, Germany; 2 Institute for Medical Informatics, Biometrics and Epidemiology, Ludwig-Maximilians-Universität München, Munich, Germany; 3 Center for International Health, Ludwig - Maximilians- Universität München, Munich, Germany; 4 International Center for Diarrhoeal Diseases Research Bangladesh (ICDDR, B), Mohakhali, Dhaka 1212 Bangladesh; 5 The George Institute for Global Health, University of Sydney, Sydney, Australia; 6 Westmead Hospital, Sydney, Australia; 7 Liverpool School of Tropical Medicine, Liverpool, UK; 8 Johns Hopkins Bloomberg School of Public Health, Baltimore, USA; 9 Diabetes Research Group, Medizinische Klinik IV, Klinikum der Universität München, München, Germany; 10 German Center for Diabetes Research (DZD), Neuherberg, Germany; 11 Clinical Cooperation Group Type 2 Diabetes, Helmholtz Zentrum München, Neuherberg, Germany

**Keywords:** Diabetes, Complications, Quality of life, Bangladesh, EQ-5D, Case control study

## Abstract

**Background:**

Little is known about the association between diabetes and health related quality of life (HRQL) in lower-middle income countries. This study aimed to investigate HRQL among individuals with and without diabetes in Bangladesh.

**Methods:**

The analysis is based on data of a case-control study, including 591 patients with type 2 diabetes (cases) who attended an outpatient unit of a hospital in Dhaka and 591 age -and sex-matched individuals without diabetes (controls). Information about socio-demographic characteristics, health conditions, and HRQL were assessed in a structured interview. HRQL was measured with the EuroQol (EQ) visual analogue scale (VAS) and the EQ five-dimensional (5D) descriptive system. The association between diabetes status and quality of life was examined using multiple linear and logistic regression models.

**Results:**

Mean EQ-VAS score of patients with diabetes was 11.5 points lower (95 %-CI: −13.5, −9.6) compared to controls without diabetes. Patients with diabetes were more likely to report problems in all EQ-5D dimensions than controls, with the largest effect observed in the dimensions ‘self-care’ (*OR* = 5.9; 95 %-CI: 2.9, 11.8) and ‘mobility’ (*OR* = 4.5; 95 %-CI: 3.0, −6.6). In patients with diabetes, male gender, high education, and high-income were associated with higher VAS score and diabetes duration and foot ulcer associated with lower VAS scores. Other diabetes-related complications were not significantly associated with HRQL.

**Conclusions:**

Our findings suggest that the impact of diabetes on HRQL in the Bangladeshi population is much higher than what is known from western populations and that unlike in western populations comorbidities/complications are not the driving factor for this effect.

## Background

Diabetes is a major health problem all over the world that leads to severe complications and disability [1]. Eighty percent of the world’s diabetic population lives in low- and middle-income countries. South Asia is one of the most affected regions [2] and with a prevalence of around 9.7 %, Bangladesh is the country with the second largest number of adults with diabetes in South Asia showing increasing trend in both urban and rural areas [3, 4]. Understanding the burden of diabetes in the Bangladeshi population is essential for decision making and resource allocation in the national healthcare system. Health-related quality of life (HRQL) is an important patient-reported outcome that allows policy makers to understand the burden of diabetes. HRQL comprehensively describes the patient’s health status comprising physical, mental, emotional and social wellbeing [5]. The EQ-5D is a preference-based HRQL instrument that has been used widely in diabetes research -- particularly preferred because of its simplicity and reliability [6]. Assessment of HRQL using generic instruments like the EQ-5D allows burden of disease comparisons across a broad spectrum of diseases and indications.

Several studies, mainly from high-and middle-income countries, have described the negative impact of diabetes on HRQL. It has been found that particularly individuals with macro-vascular complications such as stroke and ischemic heart disease often report substantial deteriorations in HRQL [7–9]. Moreover, a comprehensive review on this topic suggested a strong association between the number and severity of complications with worsening quality of life [10]. Other important determinants that have been reported were patient’s awareness that they have developed diabetes [11], insulin therapy [12], obesity [12], and fear of hypoglycemic events [13].

Even though a great proportion of people with diabetes live in South Asia, there is little evidence on the effect of diabetes on quality of life in this setting, especially in Bangladesh. The effect might be different to what is already known from western populations, as in Asia, people live in a different socio-economic context, and develop diabetes at younger age and lower BMI thresholds [14–17]. South Asians also tend to have a greater risk of developing many diabetes-related complications such as coronary artery disease (CAD), peripheral artery disease (PAD), retinopathy, nephropathy, and depression [18–20]. Given the high prevalence of diabetes and diabetic complications in South Asian populations, there is urgent need to assess and understand the burden of diabetes in terms of reduced HRQL.

The objective of this study was therefore to estimate the impact of type 2 diabetes on HRQL using data from a large case-control study from Bangladesh. In an exploratory analysis, we further examined determinants of HRQL among diabetes patients only.

## Methods

### Study design

The data of this study originates from a case-control study comprising 591 patients with diabetes and 591 people without diabetes. Non-specialized and specialized diabetes care in Bangladesh is predominantly delivered in diabetes centers or outpatient care centers. Both cases and controls were recruited in the Out-Patient Department (OPD) of the Bangladesh Institute of Health Science (BIHS) Hospital, a tertiary hospital in Dhaka, between January and July 2014. The BIHS OPD serves patients from various places and different socioeconomic backgrounds, and has one of the largest diabetic Out-Patient Department (OPD) turnover in the world under a single roof.

Individuals coming to the OPD were included as ‘cases’ if they were diagnosed with type 2 diabetes according to WHO criteria [21], willing to participate in the study by providing necessary measurements, and provide written informed consent. Exclusion criteria were age younger than 20 years, patients with serious co-morbid conditions, eg diabetic episodes, that require immediate hospitalization, patients having mental illness, or those who were unable to provide written consent.

Controls were recruited within 48 h after recruiting index cases and were either visitors of the hospital or people who live in the same geographical area as cases. Eligible were individuals of the same sex and similar age (within 5-years range) as cases, without a diabetes diagnosis, who agreed to participate by providing written consent.

### Data collection and instruments

Data were collected by a study team consisting of one study physician, one research officer, and three research assistants. The team underwent a 4 weeks training in which they were taught about the study protocol and research ethics and trained to perform the physical measurements and interviews. Information on socioeconomic status, history of diseases, diabetes-related conditions and medications, as well as HRQL were collected through face-to-face interviews using a structured questionnaire. Biomedical and anthropometric information were assessed in a standardized examination. Details on the study design, the protocol and data collection have been described elsewhere [22].

### Health related quality of life

HRQL was assessed using the Bengali translated version of EuroQol 5 dimensions (EQ-5D) instrument. The EQ-5D is a generic index instrument that is applicable to a wide range of health condition. It comprises two parts: the EQ-5D descriptive system and the EQ visual analogue scale (EQ-VAS). The EQ-5D descriptive system measures five health domains including mobility, self-care, usual activities, pain/discomfort, and anxiety/depression [23]. In the used 3 level version (EQ-5D-3 L), for each of those health domains there are 3 responses level, ie ‘no problems’, ‘moderate problems’, and ‘extreme problems’. If a scoring algorithm, ie a reference value set, is available the 243 resulting health states can be converted into a single utility value, the EQ-5D index score. If no reference value set is available, as it is the case for the Bangladeshi population, a single item analysis is possible. The EQ-VAS is a vertical Visual Analog Scale with a range from 0 (worst imaginable health state) to 100 (best imaginable health state) scale. Both the EQ-5D and the EQ-VAS evaluate the health status of respondents on the day of survey.

### Socio-demographics factors and comorbidities

Three sets of covariates were defined. The first set includes the sociodemographic and life-style factors age (continuous), gender, education (no education, primary education, secondary education, higher secondary education and above), income (categorized in quartiles), marital status (married, not married), employment status (employed, not employed), smoking status (currently smoking, not smoking) and weight status (underweight/normal weight – body mass index (BMI) < 25, overweight – 25 ≤ BMI < 30, obese-BMI ≥ 30).

The second set of covariates includes the binary variables heart disease (angina, myocardial infarction, heart failure or coronary artery disease), kidney disease (kidney failure, nephropathy or chronic kidney disease), neurological disease (stroke, transient ischemic attack or peripheral neuropathy) and eye problem (retinopathy, visual acuity or cataract).

The third set of covariates includes variables that are only important for patients with diabetes, namely diabetes duration and the presence of a foot ulcer. Selection of determinants and covariates was based on evidence from the literature and availability of information assessed.

### Data Analysis

The characteristics of the study population were described using descriptive statistics of means, standard deviations, frequencies and proportions.

To analyze the association between diabetes and EQ-VAS scores, we fitted a multiple linear regression model. To examine the relationship between diabetes and EQ-5D, the five dimensions that originally have three ordinal levels (no, moderate, extreme problems) were dichotomized to the categories ‘no problem’ and ‘moderate or extreme problem’ and a multiple logistic regressions model was applied. Linear and logistic regression models were successively adjusted for the first (socio-demographics) and then second set of covariates (comorbidities). Interaction effects between diabetes and variables from the covariate set 1 were tested. To explore the determinants of quality of life in patients with diabetes, in a second step, we applied a linear regression model in which we regressed the socio-demographics (covariate set 1), comorbidities (covariate set 2) and diabetes-related factors (covariate set 3) on the EQ-VAS scores in cases only.

Data were complete for all variables, except for “income” (*n* = 98 missing). To avoid shrinkage of data, missing values were imputed with a single regression-based imputation method (PROC MI). All analyses were performed using the PROC GLM and PROC MI statements in SAS 9.3 (SAS Institute Inc., Cary, NC, USA).

## Results

Table 1 shows the characteristics of the study population. Cases and controls did not differ significantly in terms of gender, BMI, and income, however, there was a small (0.8 years) but statistical significant difference in the matching criterion age, which probably results from the frequency matching using 5-year age bands. Patients with diabetes were more likely to be unemployed, suffered more often from cardiovascular, nephrological, neurological and eye-related diseases and were less likely to smoke or to be married. In cases, duration of diabetes averaged 7.7 years and 50 % of patients reported to suffer from at least one comorbidity or complication.

**Table 1:** Characteristics of study participants

Variables	Diabetic subjects (*n* = 591)	Non-diabetic subjects (*n* = 591)	*p*-value
Demographics			
Age; mean [SD]	51.3 [11.6]	49.5 [11.1]	0.0043*
Sex			1.000
Male; n (%)	255 (43.1)	255 (43.1)	
Female; n (%)	336 (56.9)	336 (56.9)	
BMI; mean [SD]	26.4 [3.9]	26.0 [7.0]	0.217*
Normal-underweight; n (%)	229 (38.7)	253 (42.8)	
Overweight; n (%)	274 (46.4)	271 (45.9)	
Obese; n (%)	88 (14.9)	67 (11.3)	
Education years; mean [SD]	7.8 [5.3]	9.1 [5.2]	<.0001*
No education; n (%)	116 (19.6)	76 (12.9)	
Primary education; n (%)	103 (17.4)	96 (16.2)	
Secondary education; n (%)	190 (32.2)	178 (30.1)	
Higher secondary and above; n (%)	182 (30.8)	241 (40.8)	
Income taka; mean [SD]	16605 [17096]	16629 [18825]	0.9827 *
Married; n (%)	476 (80.5)	517 (87.5)	0.0011**
Unemployed/retired; n (%)	91 (15.4)	49 (8.3)	0.0002 **
Cigarette smoking; n (%)	114 (19.3)	144 (24.4)	0.0346 **
Chronic conditions			
Cardiovascular disease; n (%)	59 (10.0)	20 (3.4)	<.0001**
Kidney disease; n (%)	53 (9.0)	14 (2.4)	<.0001**
Neurological disease; n (%)	35 (6.0)	11 (1.9)	0.0003**
Eye disease; n (%)	355 (60.1)	225 (38.1)	<.0001**
Foot ulcer; n (%)	20 (3.4)	0 (0.0)	<0.001**
Number of complications;		-	<0.001**
0; n (%)	191 (32.3)	352 (59.6)	
1; n (%)	298 (50.4)	−212 (35.9)	
> 1; n (%)	102 (17.3)	−27 (4.6)	
Diabetes duration; mean [SD]	7.7 [7.2]	-	
< 5 years; n (%)	252 (42.6)	-	
5–10 years; n (%)	148 (25.1)	-	
> 10 years; n (%)	191 (32.3)	-	

*p-values from *t*-test; **p-values from Chi-square test

### Association between diabetes and EQ-VAS

The unadjusted EQ-VAS mean in patients with diabetes was 69.0 (*SD* = 19.2), compared to 81.5 (*SD* = 15.3) in controls without diabetes. Table 2 depicts the results on the association between diabetes and HRQL. The first model, adjusted for sociodemographic variables explains 18.4 % of the variance and shows that the adjusted EQ-VAS mean in patients with diabetes was 11.5 points lower than in people without diabetes [95 % CI = −13.5; −9.6]. After adjustment for comorbidities R^2^ improved to 19.2 %, yet diabetes was still by far the strongest predictor for HRQL (*β* = −10.7 [95 % CI = −12.7; −8.6]). Interaction effects were only found for the factor unemployed/retired (*p* = 0.001). In individuals who reported to be retired/unemployed the impact of diabetes was much smaller (− 2.6 points) than in the working population (−12.6 points) (Appendix).

**Table 2:** Regression coefficients from multiple linear regression analysis on the association between diabetes status and EQ-VAS score

	Basic model (R^2^ = 0.184)		Extended model (R^2^ = 0.192)	
Covariates	β-coefficients [95 % CI]	*p*-value	β-coefficients [95 % CI]	*p*-value
Intercept	79.3 [73.0; 85.6]	<.0001	78.1 [71.8; 84.5]	<.0001
Diabetes	−11.5 [−13.5; −9.6]*	<.0001	−10.7 [−12.7; −8.6]*	<.0001
Age	−0.2 [−0.3; −0.1]*	0.0009	−0.1 [−0.2; 0.0]*	0.0142
Male (ref = female)	4.7 [2.4; 7.0]*	<.0001	4.6 [2.3; 6.9]*	<.0001
BMI (ref = <25)				
Overweight (25–30)	0.9 [−1.2; 3.0]	0.392	1.0 [−1.1; 3.0]	0.3619
Obese (>30)	−1.1 [−4.2; 2.0]	0.4865	−0.9 [−4.0; 2.2]	0.5596
Education (ref = no formal education)				
Primary	3.4 [0.1; 6.8]*	0.0462	3.7 [0.3; 7.0]*	0.0333
Secondary	4.4 [1.4; 7.5]*	0.0044	4.8 [1.7; 7.9]*	0.0021
Higher secondary and above	7.4 [4.1; 10.6]*	<.0001	7.7 [4.4; 11.0]*	<.0001
Income (ref = Q1)				
Q2	2.4 [−0.4; 5.1]	0.0875	2.3 [−0.4; 5.1]	0.0943
Q3	2.3 [−0.5; 5.1]	0.1079	2.4 [−0.4; 5.2]	0.0934
Q4	5.0 [2.1; 7.9]*	0.0008	5.1 [2.2; 8.0]*	0.0006
Married (vs single)	0.8 [−1.9; 3.5]	0.5763	0.7 [−2.0; 3.3]	0.6334
Unemployed/retired	−0.7 (−4.1; 2.7]	0.6743	−0.7 [−4.1; 2.7]	0.6972
Smoking	0.1 [−2.4; 2.5]	0.9459	0.3 [−2.2; 2.7]	0.8398
Comorbodities				
CVD	-		−2.0 [−6.0; 2.0]	0.3269
Kidney_disease	-		−2.6 [−6.8; 1.6]	0.2302
Neuro_disease	-		−4.3 [−9.4; 0.7]	0.0935
Eye_problem	-		−2.1 [−4.1; 0.0]*	0.0479

Results from multiple linear regression based on *n* = 591 cases and *n* = 591 controls; *p-value <0.05

### Association between diabetes and dimensions of the EQ-5D

**Fig. 1:**
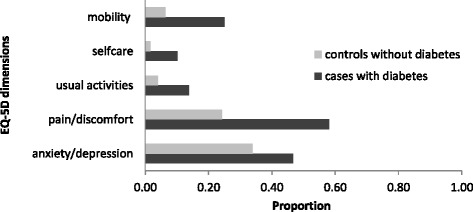
Proportion of patients reporting moderate or severe problems in the EQ-5D dimensions

Figure 1 descriptively illustrates the percentage of individuals with and without diabetes who report moderate or severe problems in the EQ-5D dimensions. Most problems were reported in the dimensions pain/discomfort (cases 58 %, controls 24 %, difference 25 %) and anxiety/depression (cases 47 %, controls 34 %, difference 13 %) followed by mobility (cases 25 %, controls 6 %, difference 19 %), usual activities (cases 14 %, controls 4 %, difference 10 %) and self-care (cases 10 %, controls 2 %, difference 8 %). Table 3 shows the adjusted odds ratios (ORs) of reporting moderate or severe problems within each of the EQ-5D dimensions in cases with diabetes compared to controls without diabetes. The adjusted OR estimates vary between 1.6 and 5.8 in the basic model. In the comorbidity-adjusted model ORs were slightly lower. In contrast to the absolute differences in proportion of reported problems, the largest relative effect was observed for the dimensions self-care (*OR* = 5.8).

**Table 3:** Adjusted odds ratios (ORs) of reporting moderate or severe problems within each of the EQ-5D dimensions in patients with diabetes compared to people without diabetes

EQ-5D dimensions	Model 1	Model 2
	OR (95 % CI)	OR2 (95 % CI)
Mobility	4.5 [3.0–6.6]	3.7 [2.5–5.6]
Self-care	5.9 [2.9–11.8]	5.2 [2.6–10.6]
Usual activities	3.3 [2.0–5.3]	3.0 [1.8–5.0]
Pain/discomfort	4.0 [3.1–5.2]	3.5 [2.7–4.6]
Anxiety/depression	1.7 [1.3–2.1]	1.6 [1.2–2.0]

Results based on multiple linear logistic regression models based on *n* = 591 cases and *n* = 591 controls. Model 1: adjusted for covariate set 1 (socio-demographics)

Model 2: adjusted for covariate set 1 (socio-demographics) and set 2 (comorbidities)

All *p*-values <0.001

### Determinants of EQ-VAS in patients with diabetes

Determinants of HRQL in patients with diabetes are illustrated in Table 4. The full model explained 10.8 % of the variance. HRQL was positively associated with male gender (*β* = +4.2), highest education level (*β* = +6.4), and belonging to the highest income quartile (*β* = +5.7). Compared to patients with a diabetes duration <5 years, those with a duration of 5–10 years and those with a duration of >10 years reported 4.7 (*p* = 0.02) and 6.3 (*p* < 0.001) point lower VAS scores. Neither weight status nor comorbidities such as heart disease, neurological disease, and renal disease and eye problems were significantly associated with EQ-VAS scores. However, diabetic foot ulcer had a large negative impact on HRQL (*β* = −13.5 [95 % CI = −21.9; −5.1]).

**Table 4:** Regression coefficients from a multiple linear regression analysis model analyzing determinates of EQ-VAS score

Parameter	β-coefficients [95 % CI]	*P*-value
Intercept	63.1 [57.5; 68.7]	<.0001
Male (ref = female)	4.2* [0.5; 7.9]	0.0281
BMI (ref = normal)		
Overweight	1.6 [−1.8; 4.9]	0.3586
Obese	1.4 [−3.4; 6.2]	0.5633
Education (ref = no formal education)		
Primary	2.4 [−2.6; 7.4]	0.3466
Secondary	3.6 [−0.8; 8.0]	0.1113
Higher secondary and above	6.4* [1.5; 11.4]	0.0113
Income (ref = Q1)		
Q2	2.8 [−1.5; 7.1]	0.1991
Q3	3.4 [−1.0; 7.9]	0.1265
Q4	5.7* [1.3; 10.1]	0.0113
Married	0.6 [−3.4; 4.5]	0.7766
Unemployed/retired	3.2 [−1.5; 8.0]	0.1846
smoking	1.0 [−2.9; 4.9]	0.6167
Diabetes duration (ref = <5 years)		
5–10 years	−4.7* [−8.5; −0.8]	0.0180
> 10 years	−6.3*[−9.9; −2.6]	0.0009
Comorbidities		
Cardiovascular diseases	−2.0 [−7.2; 3.2]	0.4490
Kidney diseases	−1.4 [−6.8; 4.0]	0.6017
Neurological diseases	−4.5 [−11.0; 1.9]	0.1691
Eye problems	−0.6 [−3.8; 2.6]	0.7195
Foot ulcer	−13.5*[−21.9; −5.1]	0.0017

R^2^ of the model = 0.108; *p-value <0.05

## Discussion

HRQL is an important patient-reported outcome that helps policy makers to understand the burden of diseases. There is a lack of evidence concerning the burden of diabetes on quality of life among South Asian populations. To the best of our knowledge, this study is the first to analyze the association between diabetes and HRQL in Bangladesh. Our findings suggest that the impact of diabetes on HRQL in the Bangladeshi population is much higher than what is known from western populations and that unlike in western populations comorbidities/complications are not the driving factor for this effect [24–28].

The results of this study show that patients with diabetes report lower VAS-scores than controls without diabetes. Qualitatively, this finding from this case-control study is in line with results from population-based studies from other parts of the world; however, the magnitude of the effect is much bigger in the Bangladeshi population. With a decrement of almost 12 points on the VAS the effect is around twice as big as what has been found in studies in the US, Germany, China and Korea in which quality of life decrements for diabetes averaged 5–7 points on the VAS [24–28].

Comorbidities were much more prevalent in cases, as diabetes is a strong independent risk factor for many of those. Though, the strong effect of diabetes on HRQL was not or only weakly mediated by self-reported micro- and macrovascular comorbidities as regression coefficients did not significantly change after adjustment for comorbidities. This is in contrast to studies from western countries which showed that micro- and macrovascular complications have a huge impact on HRQL in both patients with and without diabetes and are therefore the main causes for lower HRQL values in patients with diabetes [8, 12, 24, 29].

A recent study on the same data showed that out of pocket payments for medication and treatment are for example much higher than in China or other countries [30]. In parallel to the economic burden, this study shows that also the diabetes associated HRQL decrement is much higher than observed in other countries and populations. The reason for this finding remains unknown. It might be possible that the high out of pockets costs for diabetes medications and time resources spend for the management of the disease put individuals under financial pressure, resulting in psychologic and physical stress that translates into lower perceived HRQL. The found discrepancy in the magnitude of effect size could also be partly related to other issues: Participants in this study were slightly younger than those in the other mentioned studies and it is known that the difference in HRQL between patients with and without diabetes diminishes with age [24–28, 31]. Further, population-based HRQL studies often rely on self-reports of the diabetes status and therefore the found associations might be diluted. This is unlikely to have happened in this case-control study, as objectively measured WHO criteria for diabetes were applied. Finally, compared to population-based surveys both cases and controls were recruited in a tertiary hospital OPD. Although diabetes care in Bangladesh is predominantly delivered in such settings, it might be possible that patients visiting the hospital OPD are ‘more severe’ cases. However, as the prevalence of diabetic complications is not higher than those known from other population-based studies on routinely treated diabetes patients, this bias is expected to be rather small [32].

The reason for the low predictive value of comorbidities and complications compared to the diabetes status remains unknown. Whereas it is possible that people from Bangladesh simply value factors like renal or cardiovascular comorbidities less burdensome than in other parts of the world, discrepancies could be also related to unknown differences in assessment methods. In our study, for example, information on comorbidities were based on self-reports and various conditions were summarized in the provided answer categories.

Besides these mentioned discrepancies, our findings are generally consistent with those of previous studies from Western or other Asians countries. In our study, being male, having higher education level and belonging to the highest income group, are positively associated with HRQL, whilst older age is associated with lower quality of life. This is accordance with the literature and illustrates that particularly females and people with low socioeconomic status report lower HRQL scores [9, 12, 33, 34]. With pain/discomfort being the most frequently reported problem the results of this study are in line with other studies from various populations [7, 35–38]. In addition, as reported in previous studies we found that diabetic foot ulcer was associated with a very high HRQL decrement [36, 39]. These issues indicate that neuropathic problems in legs/feet and management of the legs are a core problem that should be addressed in the Bangladeshi population. Regular check-up of feet, screening for neuropathy, and adequate patient self-management education could be cost-effective strategies in this context.

The strengths of this study are the case-control design, the large sample size of diabetes patients, the unbiased classification of diabetes and the standardized assessment of HRQL with the EQ-5D descriptive system and the EQ-VAS. Furthermore, the stepwise adjustment for sociodemographic factors and comorbidities and the analysis of both, the impact of diabetes on HRQL, and the determinants of HRQL in patients with diabetes enhances the understanding for potentially underlying mechanisms in a high level of detail.

Yet, there are a few limitations that need to be considered when interpreting the results of the study. Firstly, all co-morbid conditions in this study are self-reported-hence misclassifications issues are likely to have had occurred. We further did not take into account the time of occurrence of comorbidities and some might already be cured by the time this study was conducted. In addition, it has to be acknowledged that this study compares quality of life of patients receiving care from a tertiary OHD with the quality of life of people without diabetes. The results of this study on the impact of diabetes on HRQL are therefore only generalizable to people who are aware of their disease, and who are under regular or sporadic care. This is a limitation, as previous studies indicated that only around 56 % of patients are aware of their disease and only 40 %, ie around 71 % of those who are aware of their disease, receive regular diabetes care [40]. Another minor limitation that has to be acknowledged is that potential cases or controls that require hospitalization were not considered to be eligible for the study. This explicit exclusion criterion leads to a minor underrepresentation of cases with severe episodes.

Finally, although the EQ-5D index score is known to be a valid and a reliable generic instrument to assess HRQL [6, 41], the results of our main analysis rely primarily on the less frequently used EQ-VAS. The reason for this is that there is no population-based Bangladeshi value set/scoring algorithm available that allows calculating a country-specific single utility score. Whereas estimations on basis of utility values describe the burden of a disease from a societal perspective, analyses on basis of VAS scores describe the burden of the disease from the perspective of the examined population, ie from the perspective of patients with diabetes. Furthermore, in contrast to EQ-5D index scores which are indirectly derived via the methods time trade-off or standard gamble, the valuations via the VAS do neither express preferences nor are they made under conditions of uncertainty. Therefore, VAS scores do not fulfill the assumptions of expected utility theory and cannot be interpreted as utilities needed as input parameters for decision analytic models.

## Conclusion

The burden of diabetes in terms of lower HRQL in Bangladesh is substantial and much larger as what has been found in other Asian, European or North American countries. Female gender, low education, low income a long diabetes duration and presence of diabetic foot ulcer were significant predictors for reduced HRQL in patients with type 2 diabetes. Secondary preventive efforts are needed and socioeconomic boundaries for treatment should be lowered to diminish the burden of diabetes and diabetic complications such as foot ulcer.

## Appendix

**Table 5:** Regression coefficients from multiple linear regression analysis on the association between diabetes status and EQ-VAS score including an interaction-term ‘diabetes*unemployed/retired’

Basic model (R^2^ = 0.192)		
Covariates	β-coefficients [95 % CI]	*p*-value
Intercept	80.4 [74.1; 86.7]	<.0001
Diabetes (yes)	−12.7 [−14.8; −10.6]	<.0001
Age	−0.2 [−0.3; −0.1]	0.0006
Male (ref = female)	4.7 [2.4; 7]	<.0001
BMI (ref = <25)		
Overweight (25–30)	0.9 [−1.2; 2.9]	0.4168
Obese (>30)	−1 [−4.1; 2.1]	0.5117
Education (ref = no formal education)		
Primary	3.3 [−0.1; 6.6]	0.0536
Secondary	4.5 [1.4; 7.5]	0.0039
Higher secondary and above	7.2 [4; 10.5]	<.0001
Income (ref = Q1)		
Q2	2.4 [−0.3; 5.1]	0.0834
Q3	2.2 [−0.6; 5]	0.1240
Q4	5.1 [2.2; 8]	0.0006
Married (vs single)	0.5 [−2.2; 3.2]	0.7083
Unemployed/retired	−7.0 [−12.1; −1.9]	0.0075
Smoking	0.1 [−2.3; 2.5]	0.9303
Diabetes*unemployed/retired	10.1 [3.9; 16.3]	0.0014

Results from multiple linear regression based on *n* = 591 cases and *n* = 591 controls; * *p*-value <0.05
